# The endoplasmic reticulum stress and the unfolded protein response in kidney disease: Implications for vascular growth factors

**DOI:** 10.1111/jcmm.15999

**Published:** 2020-10-16

**Authors:** Carlo Alberto Ricciardi, Luigi Gnudi

**Affiliations:** ^1^ King’s College of London Faculty of Life Sciences & Medicine School of Cardiovascular Medicine & Sciences Section Vascular Biology and Inflammation British Heart Foundation Centre for Research Excellence London UK

**Keywords:** acute kidney injury, chronic kidney disease, Endoplasmic reticulum stress, unfolded protein response, vascular growth factors

## Abstract

Acute kidney injury (AKI) and chronic kidney disease (CKD) represent an important challenge for healthcare providers. The identification of new biomarkers/pharmacological targets for kidney disease is required for the development of more effective therapies. Several studies have shown the importance of the endoplasmic reticulum (ER) stress in the pathophysiology of AKI and CKD. ER is a cellular organelle devolved to protein biosynthesis and maturation, and cellular detoxification processes which are activated in response to an insult. This review aimed to dissect the cellular response to ER stress which manifests with activation of the unfolded protein response (UPR) with its major branches, namely PERK, IRE1α, ATF6 and the interplay between ER and mitochondria in the pathophysiology of kidney disease. Further, we will discuss the relationship between mediators of renal injury (with specific focus on vascular growth factors) and ER stress and UPR in the pathophysiology of both AKI and CKD with the aim to propose potential new targets for treatment for kidney disease.

## 1 INTRODUCTION

Acute kidney injury (AKI) is characterized by the rapid loss of renal function which, at times, results in partial or full recovery. AKI is an independent risk factor for chronic kidney disease (CKD) as well as for end‐stage renal disease (ESRD).^1^, ^2^ CKD is characterized by a slow and progressive decline in renal function often secondary to hypertension and/or diabetic mellitus. Both AKI and CKD cases are associated with elevated cardiovascular morbidity and mortality.^3^, ^4^, ^5^, ^6^, ^7^

AKI prevalence in United States ranges between 1% and 66%, a variation that can be explained by lack of standardized AKI classification and differences between populations.^8^ CKD, conversely, is a growing pathology, with a prevalence around 15% (https://www.usrds.org/2019/view/Default.aspx), that is reaching epidemic proportions^9^ and appears to be mainly driven by an increase in the diabetic population.^10^

## 2 ENDOPLASMIC RETICULUM, GOLGI APPARATUS AND MITOCHONDRIAL NETWORKS

The endoplasmic reticulum (ER) is a continuum of membranes with tubular and vesicular shape within the cytoplasm of eukaryotic cells. The ER is constituted by the rough ER, which contains ribosomes, and smooth ER, characterized by lacks of ribosomes.^11^

In eukaryotic cells, the rough ER has a pivotal function in protein biosynthesis which serves as a checkpoint for the secretory pathway and for protein synthesis/maturation (folding) within the cell.^12^ The smooth ER is involved in carbohydrate metabolism, drug detoxification and calcium storage.^13^, ^14^

The ER could be considered as a signalling platform that responds to stimuli from in and outside the cells, with the aim of maintaining cellular functions and cell survival. The ER is the primary site for synthesis and folding of secreted and membrane‐bound proteins. Proteins are translocated into ER lumen in an unfolded state and require protein chaperones and catalysts of protein folding to assist in proper folding. Properly folded proteins traffic from the ER to the Golgi apparatus; misfolded proteins are targeted to degradation.

A proper protein folding is required to create functional proteins able to execute their function. The ER process that rectifies abnormally folded proteins which need to be physiologically replaced/restored or have been damaged by external insults is defined as 'unfolded protein response' (UPR) characterized by three major pathways: PERK, XBP‐1 and ATF6.^15^ UPR provides an adaptive mechanism by which cells can augment protein folding and processing capacities of the ER. If protein misfolding is not resolved, the UPR triggers an apoptotic cascade.^16^

The Golgi apparatus is a dynamic organelle constituted by several membranes closely linked with the ER. The Golgi has a crucial function in regulating the trafficking of proteins within the cell, and processing newly synthesized polypeptides, secretory proteins and lipids.^17^ Proteins and lipids are delivered from ER to the Golgi apparatus and to the plasma membrane with the auxilium of molecular tags that direct them to their cellular destinations.^18^ Conversely, misfolded proteins that escape from the ER are processed by the Golgi or delivered to the lysosome/vacuole for degradation.^19^

The mitochondria are double‐membrane‐bound organelles with a highly specialized structure involved in cellular respiration and energy homoeostasis.^20^

ER and mitochondria communicate through contact points known as mitochondria‐associated membranes (MAMs).^21^ MAMs should not be seen as a static link between ER and mitochondria. On the contrary, the MAMs is represented by a variable collection of proteins that regulate signals between the ER and mitochondria according to the cells’ needs.^22^

MAMs are implicated in the calcium transfer from the ER to mitochondria and maintain adequate mitochondria bioenergetics and lipid synthesis and mitochondrial shape and motility. MAMs are essential for the transfer of stress signals from the ER to mitochondria via the activation of UPR.^23^ Several MAM connectors can modulate the UPR; of these, mitofusin‐2 protein (Mfn2) not only supports mitochondria‐ER physical interaction,^24^ but also participates in the cellular response to ER stress by suppressing PERK activation through direct interaction. In condition of ER stress, loss of Mfn2‐PERK interaction in Mfn2‐deficient cells results in the activation of all three UPR branches (PERK, XBP‐1 and ATF6) resulting in reduced cell apoptosis.^25^

Similarly, chaperones, proteins that assist the conformational folding/unfolding and the assembly/disassembly of macromolecular structures in the ER, are important components of MAMs and favour the ER‐mitochondria interaction, and contribute to the regulation of different cellular functions including calcium signalling, energy metabolism and cell survival.^26^

The interconnectivity between the ER, the Golgi apparatus and mitochondria has the role to sustain a pro‐survival cellular response to external perturbations^26^ such as excess of substrates (eg glucose, lipids), oxidative stress, iron imbalance, viral infections and hypoxia (known as important causes of ER stress).^27^

When a pathological insult is sustained, and cell survival is compromised, the ER‐UPR participates in the initiation of apoptosis by at least two main mechanisms. Firstly, apoptosis could be triggered by a progressive accumulation of misfolded protein in the ER when protein ubiquitination and degradation mechanism are overcome. Accumulation of misfolded proteins would trigger a sustained UPR activation and activation of cellular apoptosis mainly via C/EBP homologous protein (CHOP) and IRE1 pathway activation.^26^, ^28^ Secondly, apoptosis can be induced through the calcium signalling. ER stress‐induced apoptosis is characterized by calcium release from the ER with concomitant increase in calcium within the mitochondria. In mitochondria, calcium leads to inner mitochondrial membrane depolarization and activation of pro‐apoptotic caspase‐mediated pathways. ^26^, ^29^

## 3 ER‐MITOCHONDRIA INTERPLAY IN RENAL DISEASE

The kidney mitochondrial content is second only to the heart and is crucial for the kidney physiology and pathophysiology of diseases. Mitochondrial dysfunction is universally recognized as a mechanism involved in both AKI and CKD.^30^

As discussed above, the communication between ER and the mitochondria has pivotal roles in cellular function such as gene transcription, calcium homoeostasis and cellular redox states.^26^

As a response to acute ER stress, UPR (via PERK) drives the dynamic regulation of the mitochondria morphology promoting stress‐induced mitochondrial hyperfusion (SIMH).^31^ SIMH is a known pro‐survival mechanism that reduces mitochondrial fragmentation and promotes mitochondrial functions, such as ATP production, to protect cells in response to acute insults.^31^

Thus, the PERK arm of the UPR regulates mitochondrial morphology during acute ER stress,^31^ confirming that UPR, during an acute perturbation as seen in AKI, has a key protective role.

Acute kidney ischaemia is, at times, not followed by organ recovery; this is often paralleled by long‐term morphological and functional damage of renal cells^32^ and by a reduction in mitochondrial number.^30^ Incomplete kidney recovery after AKI often becomes a predisposing factor towards further renal deterioration and development of CKD.^32^

It is well established that mitochondrial homoeostasis can put a brake on the progression of kidney disease and favour a renal recovery from acute insult.^33^ On the contrary in CKD, sustained ER stress drives chronic mitochondrial reactive oxygen species generation thus promoting mitochondrial dysfunction.^33^ The sustained redox imbalance as seen in CKD contributes to mitochondrial remodelling and cellular damage with no cell recovery, contrarily to what is seen in AKI (transient insult), promoting a progressive chronic renal function decline towards ESRD.^34^

It is also worth remembering that mitochondrial biogenesis (process by which cells increase mitochondrial mass)^33^ occurs after exposure of cells to external insults and plays a role in UPR activation and positive response to ER stress.^35^ Mitochondrial biogenesis requires the synthesis of nuclear and mitochondrial encoded proteins; of note, this augmentation in protein synthesis can, if sustained, disrupt cellular proteostasis by exceeding the protein‐folding capacity of the cell and contributing to ER stress and to a chronic disease process.^36^, ^37^

## 4 ER STRESS AND UPR IN KIDNEY DISEASE

ER stress has been implicated in the pathophysiology of both AKI and CKD.^38^ Protein misfolding and secondary ER stress have been described in AKI, glomerulonephritis, glomerulopathies associated with genetic mutations, and hypertensive and diabetes‐related CKD.^39^

AKI leads to the accumulation of unfolded and misfolded proteins in the ER. The accumulation of non‐functional proteins causes an altered condition of protein homoeostasis that stimulates the UPR to decrease protein translation and increase ER protein‐folding capacity, and activates pathways involved in protein degradation to restore proteostasis.^40^

In AKI, renal ischaemic injury such as renal vascular obstruction, cardiac arrest and renal ischaemia‐reperfusion injury in kidney transplantation, is the main driver of ER stress and secondary UPR activation and tissue injury.^39^, ^40^ Similarly, AKI drug‐induced nephrotoxicity (eg cisplatin induced) is characterized by a significant activation of ER stress, often associated with cell apoptosis.^40^, ^41^

Inflammation is one of the main mediators of AKI.^42^ In AKI different cytokines and chemokines are released by leucocytes and renal tubular cells. The pro‐inflammatory cytokines/chemokines interferon‐γ, interleukin‐2, interleukin‐6 and interleukin‐10, granulocyte‐macrophage colony‐stimulating factor, transforming growth factor‐β 1 (TGF‐β‐1), chemokine (C‐X‐C motif) ligand‐1, macrophage inflammatory protein‐2 and monocyte chemoattractant protein‐1 are increased in the ischaemic kidney.^42^ Limited work has described a clear link between ER stress inflammatory cytokines and UPR in the kidney; nevertheless, transcriptional regulators of inflammatory genes such as nuclear factor‐kB (NF‐kB),^43^ and cyclic adenosine monophosphate (cAMP)‐responsive element‐binding protein H (CREBH), an ER‐localized transcription factor,^44^ have been shown to initiates an acute inflammatory response after activation by ER stress.

**FIGURE 1:**
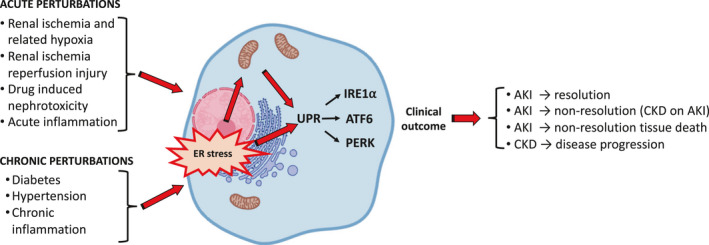
ER stress drives different UPR‐mediated cellular/tissue outcomes in AKI and CKD. Acute and chronic insults to the kidney result in a transient or sustained activation of ER stress/mitochondria and UPR, respectively. ER stress/UPR plays an important role in determining favourable/not favourable clinical outcome of a disease

It is still unclear why an episode of AKI is at times followed by a partial/full recovery of renal function or, instead, progresses towards ESRD.^7^ AKI is a manifestation of different pathological processes which, despite similar ER stress‐mediated UPR activation, can result in different outcomes often difficult to predict.^45^ Functionally, under mild‐moderate ER stress, the activation of the UPR to restore proteostasis leads to cell survival. However, in condition of excessive ER stress, the adaptive capacity of UPR is lost resulting in cell apoptosis to eliminate the irreversibly damaged cells and tissue (Figure 1).

The different degree of ER stress and UPR stimulation, that either lead to cell survival or death, should not be simply related to a positive or negative outcome. We will need to understand more and better the mechanisms implicated in directing the kidney outcome after AKI. The AKI‐related ER stress likely depends on the magnitude of the insult, the length of the perturbation and the overall condition of the patient; all these variables could contribute to patients’ response to ER stress and prognosis after an episode of AKI. There is certainly an urgent need to investigate ER stress in samples from AKI patients to understand the molecular mechanisms behind a favourable or poor outcome and plan clinical trials to test the effect of drug modulating ER stress and UPR.

AKI is regarded as a major risk factor for the development of CKD and AKI could influence CKD’s progression.^40^, ^46^ Important AKI‐related insults could result in a defective repair of the renal tubular compartment with activation of ER stress‐mediated pro‐apoptotic, pro‐inflammatory and pro‐fibrotic pathways to attempt wound repair that results in renal interstitial fibrosis which culminates in progressive loss of renal function.^40^, ^47^

Our understanding of ER stress and UPR in AKI is quite limited but ER or mitochondrial dysfunction following injury has been implicated in the alteration of mitochondria‐ER crosstalk in the kidney, an event that has been involved in the progression of AKI to CKD.^48^

In patient with diabetes mellitus and/or hypertension, chronic perturbations such as hyperglycaemia, dyslipidaemias and haemodynamic insults (elevated systemic‐hypertension and intraglomerular pressure) result in ER stress^49^, ^50^ and are important factors driving renal decline progression towards CKD and ESRD.^51^, ^52^

CKD is characterized by a condition of sustained ER stress, mitochondrial dysfunction, sustained UPR‐mediated cell death and autophagy.^39^

Importantly, chronic elevated glucose, free fatty acid and advanced glycation end products promote a sustained activation of S‐XBP1 and CHOP, which results in the apoptosis of glomerular endothelial and tubular cells.^49^, ^52^ Lipid accumulation in renal tubular cells results in mitochondrial dysfunction, increased oxidative stress and inflammation that contributes to extracellular matrix production and tubulointerstitial fibrosis.^53^ Importantly, ATF6α, a transcription factor of the UPR, regulates fatty acid metabolism and its overexpression in tubular renal cells results, via peroxisome proliferator‐activated receptor‐a (PPARα) down‐regulation, in cellular lipid accumulation with lipotoxicity‐mediated cellular apoptosis with up‐regulation of pro‐sclerotic cytokines and tubulointerstitial fibrosis.^54^

Studies conducted in experimental animal models of diabetes (streptozotocin‐induced diabetes and db/db mice) have shown a sustained activation of UPR, with increased levels of phosphorylated PERK, eIF2α and CHOP, with activation of ATF6 and caspase with secondary glomerular and tubular cells apoptosis.^55^, ^56^, ^57^ Further, mice mutant for glucose‐regulated chaperon protein‐78 (GRP78) show impaired chaperone‐mediated protective effects and develop severe tubulointerstitial lesions with ageing.^58^

The sustained UPR activation leads to up‐regulation of inflammatory and fibrotic cytokines that result in progressive tissue injury and fibrosis as a manifestation of wound repair.^59^ These processes are even worsened when AKI occurs in patients with CKD; these patients are known to have a severe prognosis and partial or full renal recovery is delayed and often absent.^60^

Chronic sustained inflammation as seen in CKD is a promoter for uncontrolled healing and tissue damage. ER stress drives inflammatory signalling that, per se, facilitates fibrotic processes.^59^ In this condition, sustained activation of the UPR pathways, such as IRE1α, results in the activation of tumour necrosis factor receptor and the pro‐inflammatory transcription factor AP‐1, which have been shown to promote the activation of pro‐inflammatory pathways, such as NF‐KB, NOD1/2 and RIP‐dependent cascades.^59^ Similarly, PERK activation and eIF2α phosphorylation increase NF‐KB stability. ER stress in macrophages supports polarization towards a pro‐inflammatory phenotype driven by NF‐KB.^59^, ^61^ with increase in pro‐inflammatory cytokines such as interleukin 1‐β and interleukin‐18. Indeed, IRE1α in macrophages has been implicated in promoting macrophages polarization towards a pro‐inflammatory rather than a non‐inflammatory pro‐sclerotic phenotype.^59^, ^62^, ^63^ Further, epithelial cells exposed to ER stress lose their characteristic and undergo an epithelial‐to‐mesenchymal transition that reprogram the cells promoting a fibrotic process that leads to tissue injury and loss of normal renal tissue architecture.^64^, ^65^

Importantly, TGF‐β‐1 is a pro‐sclerotic cytokine that plays a crucial role in ER stress activation and secondary fibrosis: in cultured renal tubular cells, TGF‐β1 induces the ER stress/UPR pathways which mediate the pro‐fibrotic processes.^59^, ^66^, ^67^

## 5 VASCULAR GROWTH FACTORS IN THE PATHOPHYSIOLOGY OF AKI AND CKD

Different vascular growth factors have been implicated in the pathophysiology of both AKI and CKD.

### 5.1 Vascular endothelial growth factor A (VEGFA)

In physiology, VEGFA is constitutively expressed in the glomeruli (podocytes) and its deletion or overexpression result in alteration of the permselective properties of the glomerular filtration barrier.^68^

In disease setting, such as AKI, VEGFA supplementation is protective for glomerular and tubulointerstitial injury in different experimental models of ischaemic renal disease^69^ and its administration helps recovery.^70^

In the initial phase of ischaemia/reperfusion, in a rat experimental model of AKI, VEGFA was initially down‐regulated^71^ and recovery was paralleled by a normalization of VEGFA expression.^72^, ^73^

Rapidly progressive glomerulonephritis characterized by acute loss of renal function is characterized by a protective up‐regulation of VEGFA^74^ as blockade of VEGFA results in worsening of the disease.^75^

In membranoproliferative glomerulonephritis, studies have demonstrated a protective VEGFA up‐regulation for the endothelium,^76^, ^77^ suggesting, again, that in acute setting VEGFA therapy could help to increase capillary repair.

In CKD, when the insult is sustained such as in diabetic nephropathy, VEGFA expression/action is up‐regulated and its inhibition has proven beneficial.^68^, ^78^ Of importance, VEGFA action should be modulated and not completely suppressed as VEGFA is essential for endothelium survival and renal function.^79^, ^80^

In immunodeficiency virus (HIV) nephropathy, a progressive condition, up‐regulation of VEGFA appears to be implicated in its pathophysiology.^81^ VEGFA up‐regulation has been observed in humans with HIV and in animal experimental models of HIV nephropathy and VEGFA inhibition has been proposed as a potential treatment.^82^, ^83^

### 5.2 Angiopoietins (Angpt)

Angpt are vascular growth factors involved in vasculogenesis and vascular repair. Angpt1, by binding to its receptor tyrosine kinase with immunoglobulin and epidermal growth factor homology domain‐2 (Tie2), stabilizes the vessel wall, while Angpt2, by either interacting with integrins or competing with Angpt1/Tie2 receptor binding (inhibiting Angpt1‐mediated Tie2 phosphorylation), promotes vessel wall destabilization and, in the presence of VEGFA, endothelial cell proliferation and new vessel formation.^68^

In physiology, Angpt1 is expressed by glomerular cells and pericytes, while Angpt2 expression is absent; increased levels of Angpt2 have been observed in glomerular diseases and appear to be correlated to adverse outcomes.^68^

In experimental model of AKI such as ischaemic kidney injury, up‐regulation of Angpt1 attenuates renal damage mainly by promoting endothelium cell survival and vascular repair while down‐regulation is associated with impaired renal recovery.^84^

In critically ill patients, Angpt1 circulating levels are associated with a lower risk of AKI; conversely, higher Angpt2 levels were associated with an increased risk for AKI.^85^ Of interest, Angpt1 therapy protects against AKI caused by endotoxemia and ischaemia‐reperfusion.^86^, ^87^

In CKD, an increased ratio of Angpt2/Angpt1 plays a role in the development and progression of glomerular disease in diabetes^88^ and correction of this unbalance has been proposed as protective.^89^, ^90^

As per AKI, a recent study proposed Angpt2 as an independent predictor of adverse renal outcome in chronic kidney disease in both the general and the diabetic population.^91^

Angpt2 levels also show a significant independent correlation with proteinuria in another progressive renal disease such as systemic lupus erythematosus.^92^

### 5.3 Fibroblast Growth Factor (FGF)

In AKI, renal ischaemia‐reperfusion injury is associated with a poor renal prognosis. FGFs, important angiogenic inducers,^93^ are up‐regulated in AKI.^94^ Mainly, FGF2 and FGF10, in averting ischaemia/reperfusion‐induced tubular cell death, have been proposed as potential future targetable pathway and more work is needed to explore their protective role in AKI.^95^

In CKD, the role of FGFs is still unclear and more studies are warranted. One of the more studied is FGF23. FGF23 has been proposed to have a protective effect on the vasculature in CKD by reducing vascular calcifications but, despite these findings, high circulating levels of FGF23 have been associated with increased cardiovascular disease independent of CKD.^96^

### 5.4 Epidermal growth factor (EGF)

EGF mediates its effects on cell growth, differentiation and apoptosis by binding to the cell surface tyrosine kinase epidermal growth factor receptor (EGFR). EGFR is expressed in the kidney, both in the glomerular and tubular compartments. EGF effects are either direct or mediated by EGF‐mediated VEGFA up‐regulation.^97^

In AKI, EGFR activation enhances renal recovery as suggested by studies in experimental animal model of AKI, where proximal tubule cell deletion of EGFR or treatment with an EGFR inhibitor resulted in impaired recovery from ischaemia‐reperfusion‐induced injury.^98^ In rapidly progressive crescentic glomerulonephritis, blockade of EGF is also beneficial.^99^

In CKD, as seen in diabetes, EGF is up‐regulated^100^ and inhibition of EGF/EGFR system attenuates renal damage in an experimental animal model of diabetes.^101^, ^102^

Similarly, in experimental model of hypertensive nephropathies, EGF/EGFR system inhibition seems to confer protection to the kidney.^103^

## 6 VASCULAR GROWTH FACTORS AND ENDOPLASMIC RETICULUM STRESS IN KIDNEY DISEASE

Chronic hypoxia plays a crucial role in the development/progression of kidney disease.^104^, ^105^ In response to hypoxia, the kidney up‐regulates angiogenic factors such as VEGFA to repair/restore the renal capillary network. In CKD, this adaptive repair mechanisms are impaired because of chronic inflammation and tissue scarring that results in progressive capillary rarefaction and kidney tissue damage.^105^

Accumulating evidence suggests that ER stress‐dependent or ER stress‐independent activation of UPR modulates vascular growth factors expression/activity and plays an important role in the maintenance and survival of endothelial cells and vasculature.^106^, ^107^ Other studies have observed that vascular growth factors, per se, can directly signal and modulate the UPR.^108^

**FIGURE 2:**
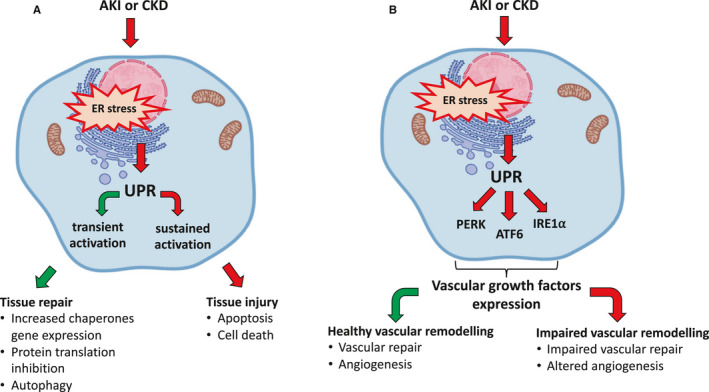
ER stress/UPR transient or sustained activation modulates tissue repair/injury and healthy/impaired vascular remodelling, respectively. A, Acute insults (AKI) stimulate a transient UPR that contributes to tissue repair. Conversely chronic insults, as seen in CKD, stimulate a sustained UPR which promotes tissue injury. B, In condition of ER stress, secondary to AKI or CKD, UPR plays a role in modulating the expression of vascular growth factors. The modulation of vascular growth factor expression drives a healthy or impaired vascular remodelling in condition of transient or sustained UPR activation, respectively

As discussed, a transient activation of the UPR mostly retains a pro‐survival effect that promotes vascular repair and new vessel formation with tissue recovery; conversely, sustained UPR activation, when tissue damage is prolonged, promotes impaired vascular remodelling cell death and tissue degeneration (Figure 2).

Therefore, modulation of ER stress/UPR may provide attractive novel strategies to ameliorate/correct altered vascular repair and anomalous angiogenesis in diseases without the need of completely blocking vascular growth factors, such as VEGFA or Angpt, known to play many essential roles in kidney tissue homoeostasis.^78^, ^109^

In a human proximal tubular epithelial cell line (HK­2 cells) and *in vivo* (in a model of renal ischaemia), UPR activation drives the expression of vascular growth factors such as VEGFA and basic fibroblast growth factor via PERK activation.^39^, ^110^ Indeed, PERK is known to regulate the pro‐angiogenic protein basic FGF^93^ expression at both the transcriptional and translational levels.^110^ Moreover, transcription factors from all three arms of the UPR (ATF4, spliced XBP‐1 and cleaved ATF6) have consensus sites on the promoter region of VEGFA and have been shown to drive its transcription.^111^, ^112^

**FIGURE 3:**
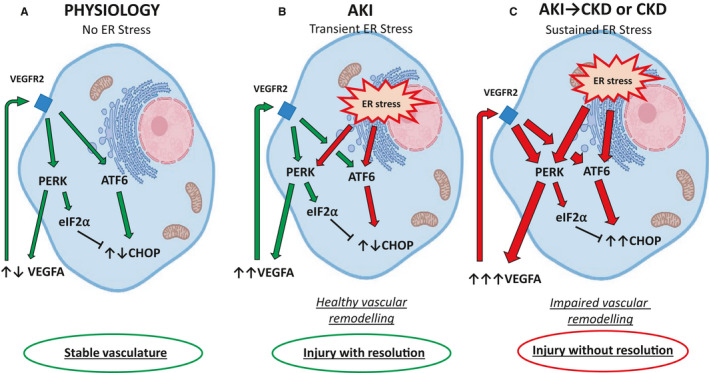
VEGFA/VEGFR2 system differentially modulates the UPR activation in condition of transient or sustained ER stress. A, In physiology, UPR is activated by the VEGF/VEGFR2 system in the absence of ER stress. The VEGF/VEGFR2 system activates both PERK and ATF6 resulting in cell survival and inhibition of the pro‐apoptotic pathway CHOP favouring a cells’ survival. B, AKI mediates (transient) ER stress and PERK and ATF6 activation, which, in turn, drive an up‐regulation of VEGFA. VEGFA, with an autocrine/paracrine mechanism, activates PERK and ATF6 via VEGFR2 activation. The balance between VEGFA/VEGFR2 system and transient UPR‐mediated PERK and ATF6 activation results in lack of stimulation of CHOP‐mediated cell apoptosis, cell survival and tissue repair. C, CKD after AKI or CKD is characterized by sustained ER stress and UPR stimulation. Sustained ER stress‐mediated UPR activation results in CHOP‐mediated cell apoptosis. The sustained stimulation of PERK up‐regulates the VEGFA/VEGFR2 system resulting in a synergistic (VEGFA/UPR) activation of CHOP and cell apoptosis resulting in tissue injury

It is also worth remembering that VEGFA signals via ATF6 and PERK. VEGFA‐mediated activation of ATF6 and PERK contributes to the survival effect of VEGFA on endothelial cells by promoting mTORC2‐mediated phosphorylation (and activation) of AKT^Ser473^.^108^ UPR pathways constitute important components of VEGFA signalling and endothelial cell survival and angiogenesis, independently of ER stress‐mediated UPR activation^108^ (**Figure** **3**).

VEGFA is crucial in the maintenance of glomerular capillaries homoeostasis^113^ and inhibitors of ER stress‐mediated UPR stimulation^114^, ^115^ suppress VEGFA causing proteinuria.^116^ These observations clearly show a close link between UPR activation and VEGFA. It is worth noting that too little VEGFA is not favourable for cell homoeostasis,^68^ a concept which seems to apply also to inhibition of adaptive UPR activation.

Other vascular growth factors such as Angpt have been implicated in vascular repair in kidney disease being Angpt1 protective for the vasculature.^68^, ^89^, ^90^, ^117^ Angpt1 attenuates ER stress‐induced cellular dysfunction and apoptosis in glomerular endothelial cells. Specifically, Angpt1 is able to prevent/ameliorate the angiotensin‐2–induced expression of UPR proteins and PERK/CHOP activation.^118^

FGFs are potent angiogenic inducers^93^ and seem to modulate ER stress and UPR. FGF1 treatment suppressed diabetes‐induced ER stress in an experimental model of diabetic nephropathy,^119^ and FGF10 significantly attenuates the apoptosis of kidney tissues in AKI caused by renal ischaemia‐reperfusion injury by attenuating UPR.^120^

EGF, also involved in angiogenesis, and its receptor EGFR activation have been involved in the development and progression of diabetic nephropathy. Inhibition of EGFR tyrosine kinase activity with erlotinib attenuates renal damage in an experimental animal model of diabetes, partly by inhibition of ER stress.^101^ Further studies have suggested a role of EGFR activation and ER stress in angiotensin‐2–mediated vascular remodelling, a phenomena independent from angiotensin‐2–mediated hypertension.^121^

## 7 CONCLUSIONS

The ER stress (external insult) and the UPR are crucial mechanisms that regulate cell survival or death. Vascular growth factors directly regulate UPR and also modulate ER stress‐mediated UPR. Blockade or activation of the UPR in disease setting will not provide a clear‐cut answer to potential treatment for both AKI and CKD. Most importantly, drugs able to modulate the UPR will be extremely important. We will need to learn to consider many variables such as the magnitude and duration of the perturbation and the health‐related conditions of the patient to be able to design the correct modulation of the UPR to result in patient benefit.

More studies are needed, and future work will certainly open new treatment avenues for the treatment of the patient with renal disease.

Funding grant: TF_001_20171120.

## 8 CONFLICT OF INTERESTS’ STATEMENT

The authors declare no conflict of interest on the topic covered.

## AUTHORS’ CONTRIBUTION

Carlo Alberto Ricciardi: Conceptualization (equal); Data curation (equal); Funding acquisition (equal); Investigation (equal); Methodology (equal); Validation (equal); Visualization (equal); Writing‐original draft (equal); Writing‐review & editing (equal). Luigi Gnudi: Conceptualization (equal); Data curation (equal); Formal analysis (equal); Funding acquisition (equal); Investigation (equal); Methodology (equal); Project administration (equal); Resources (equal); Supervision (equal); Validation (equal); Writing‐review & editing (equal).
